# The importance of community resources for breastfeeding

**DOI:** 10.1186/s13006-024-00623-2

**Published:** 2024-03-06

**Authors:** Jennifer LoCasale-Crouch, Margaret Kathleen Wallace, Timothy Heeren, Stephen Kerr, Yitong Yue, Genevieve Deeken, Khara Turnbull, Brianna Jaworski, Mayaris Cubides Mateus, Rachel Moon, Fern Robin Hauck, Ann Kellams, Eve Colson, Michael Jay Corwin

**Affiliations:** 1 School of Education, Virginia Commonwealth University, Richmond, VA USA; 2 School of Public Health, Boston University, Boston, MA USA; 3 Chobanian & Avedisian School of Medicine, Boston University, Boston, MA USA; 4 School of Education and Human Development, University of Virginia, Charlottesville, VA USA; 5 Department of Global Public Health– Global Studies, University of Virginia, Charlottesville, VA USA; 6 School of Medicine, University of Virginia, Charlottesville, VA USA; 7 School of Medicine, Washington University in St. Louis, Saint Louis, MO USA

**Keywords:** Breastfeeding, Community resources, Health behaviors, Equity

## Abstract

**Background:**

Breastfeeding has long-lasting effects on children’s cognition, behavioral, mental and physical health. Previous research shows parental characteristics (e.g., education, race/ethnicity, income level) are associated with breastfeeding initiation and duration. Further, research shows significant variation in access to community resources by race/ethnicity. It is unclear how community resources may impact breastfeeding practices and how this might intersect with maternal race/ethnicity.

**Methods:**

This study combined nationally-representative data from the Study of Attitudes and Factors Effecting Infant Care (SAFE), which surveyed US mothers immediately after the infant’s birth and at two to six months of infant age, with the Child Opportunity Index (COI) 2.0, a census tract measure of community resources associated with child development, to explore the association between community resources and breastfeeding initiation and whether this varies based on maternal race/ethnicity and country of birth. The SAFE Study used a stratified, two-stage, clustered design to obtain a nationally representative sample of mothers of infants, while oversampling Hispanic and non-Hispanic (NH) Black mothers. The SAFE study enrolled mothers who spoke English or Spanish across 32 US birth hospitals between January 2011 and March 2014.

**Results:**

After accounting for individual characteristics, mothers residing in the highest-resourced communities (compared to the lowest) had significantly greater likelihood of breastfeeding. Representation in higher-resourced communities differed by race/ethnicity. Race/ethnicity did not significantly moderate the association between community resources and breastfeeding. In examining within race/ethnic groups, however, community resources were not associated with non-US born Black and Hispanic mothers’ rates of breastfeeding, while they were with US born Black and Hispanic mothers.

**Conclusions:**

Findings suggest that even health behaviors like breastfeeding, which we often associate with individual choice, are connected to the community resources within which they are made. Study implications point to the importance of considering the impact of the contextual factors that shape health and as a potential contributor to understanding the observed race/ethnicity gap.

## Background

Infants’ healthy development depends on access to quality nutrition and protection from birth. Human milk, typically provided through breastfeeding, is recognized as the optimal source of nutrition and protection for infants. Human milk has been consistently linked to overall health and well-being, both in childhood and later into adulthood [1]. Thus, familial characteristics associated with initiation and duration of human milk feeding have been rigorously studied. In the United States (U.S.), there are large racial/ethnic disparities in breastfeeding initiation and duration that are attributed to systemic inequities, with differences of up to 40% points between racial groups [2]. For example, Black mothers are significantly less likely to breastfeed when compared to white, Latiné/Hispanic or Asian mothers, and these differences are linked to education, social status, employment, and systemic racism [3]—which all pose significant barriers to breastfeeding practices nationwide.

Parents and their infants do not exist in isolation. They are part of communities that vary in their access to, or lack of, resources that foster healthy development. Those who reside in more advantaged communities may have short-term, long-term, and even lifelong developmental benefits, including better health outcomes [4], higher educational achievements [5], and later upward social mobility [6]. Little work has examined the extent to which a community’s resources might support or inhibit breastfeeding. If community resources contribute to an individual’s ability to breastfeed, equitable approaches may be possible through policies and practices focused on the broader community that then support individuals. Thus, this study examines whether breastfeeding is associated with the availability of community resources and whether this differs based on maternal characteristics, including race, ethnicity and country of birth.

### The value of breastfeeding for child development and intersection with parental characteristics

Breastfeeding has significant and long-lasting effects on cognition, behavioral, mental and physical health in children [1, 7]. Thus, breastfeeding serves as a key support to infants in the early stages of development, setting the trajectory for optimal future development. Furthermore, exclusive breastfeeding for the first six months of infancy has been shown to improve infant immunity by reducing risks to both short and long term illnesses, resulting in benefits such as lower rates of respiratory tract infections, sudden infant death syndrome, asthma, and diabetes [8]. Exclusive breastfeeding is associated with numerous maternal benefits as well, such as a reduced risk of high blood pressure, diabetes, depression and both breast and ovarian cancer [9]. The findings of infant and maternal benefits have yielded research and policy consensus that infants should be exclusively breastfeed for approximately six months, a recommendation promoted by both the American Academy of Pediatrics and the CDC [1, 10].

Studies examining the association between parental characteristics and breastfeeding outcomes have particularly focused on mothers. Formal education is consistently related to breastfeeding practices [11, 12], with those who report lower levels of education reporting breastfeeding for shorter durations. In one study, compared to college-educated mothers, those who did not complete high school were significantly less likely to breastfeed [13]. Further, breastfeeding is more likely to occur when the mother is married, middle-high income, employed, white or Latiné/Hispanic or 30 + years old [14]. Digging deeper into the racial disparities in breastfeeding rates, Chiang and colleagues examined differences by race/ethnicity among over three million births in the 2019 National Vital Statistics System database [2]. They found that initiation of breastfeeding varied greatly among racial groups throughout the U.S., from 90.3% for Asian mothers, compared to 73.6% for Black mothers. Thus, education and race/ethnicity seem to be key factors to consider in examining breastfeeding rates.

### Consideration of social determinants in understanding breastfeeding practices

Social factors, such as marital status, employment, education, and healthcare, are also affected by the racially-disparate socioeconomic system present in the U.S [15]. Thus, the societal factors that are inequitably available across race/ethnicity may play a critical role in a parent’s ability to breastfeed. Interestingly, though, breastfeeding practices vary for Black and Latiné/Hispanic women in the U.S. when country of birth (U.S. or non-U.S.) is taken into consideration. Safon and colleagues recently found that non-U.S.-born Black mothers were seven times more likely to breastfeed than U.S.-born Black mothers [15]. In a similar study examining breastfeeding patterns among U.S. and non-U.S.-born Latiné/Hispanic mothers, Safon and colleagues found that at two to six months, compared with U.S.-born white mothers, those born in Mexico, Central America, and South America were more likely to breastfeed, and those born in the Caribbean were less likely to breastfeed [16]. This more nuanced look within race/ethnicity groups may provide insights into how future interventions might support people differently based on their unique needs and historical patterns.

### The potential role of community resources in breastfeeding practices

Living in higher resourced communities is associated with lower obesity levels, lower prevalence of the leading causes of childhood death and chronic disease, and resilience in the face of trauma [4]. There is also a significant relationship between neighborhood resources and well-being outcomes such as early school readiness and later earnings and social mobility [6, 17–20]. Further, parents who live in communities that are lower-resourced experience higher levels of stress and depression, which potentially limits their ability to be responsive to their family’s needs [21–23]. These findings persist after family- and school-level confounders are controlled for, suggesting that communities may have a unique influence on children’s development [19, 24, 25], and that the negative impacts of living in low resourced communities during early childhood may have lasting consequences. Little research, however, has explored associations between community resources and the extent to which adults engage in behaviors such as breastfeeding.

Communities and the resources therein may play a role in a parent’s capacity to breastfeed. In a qualitative study, low-income, Hispanic mothers perceived that lack of access to healthy food impacted their milk quality [26], and the authors surmised that maternal beliefs about stress, limited access to nutritious foods, and an unhealthy maternal diet might lead to decreased breastfeeding. In another study, Dinour and colleagues found that food-secure mothers were significantly more likely to breastfeed than mothers experiencing food insecurity [27]. Further, a recent study examining the impact of COVID-19 on breastfeeding demonstrated that mothers who lived in more resourced communities had more positive breastfeeding experiences during lockdown than those who lived in less resourced communities [28]. The study pointed to several community-level factors, including access to green spaces, places to get out and exercise, and fast and stable internet connections, as useful to mothers’ continued breastfeeding.

In addition, some research points to the intersection of parental characteristics and community resources in relation to breastfeeding. For example, living in a low-income community increases stress levels [29] and is closely related to mothers’ education level [30], which has been associated with breastfeeding [12]. Santiago and colleagues pointed out that neighborhood poverty could impact individuals’ mental health, which might potentially lead to decreased breastfeeding [26, 29]. Additionally, Tang and colleagues found that mothers with higher education and who were from high-income households were more likely to initiate breastfeeding than their counterparts without these resources [12]. Thus, examining the intersection of place, race and breastfeeding practices appears warranted.

### The intersection of race and community resources

While research utilizing community socioeconomic characteristics has pointed to the importance of context on individual behaviors, examining a community’s socioeconomic status alone overlooks evidence suggesting that there is considerable variability in access to resources within communities, and that this access varies by race/ethnicity [31–33]. For example, Hardy and colleagues found that nearly one-quarter of poor white children live in very low-resourced communities, while almost half live in moderate-, high- and very high-resourced communities [32]. On the other hand, close to 70% of Black children from low-income families live in very low-resourced communities. Thus, Black children from low-income families are three times more likely than white children from low-income families to be in the lowest-resourced communities, with very few (5%) having access to high- or very high-resourced ones. This finding suggests that racial disparities in breastfeeding may be due to other social determinants of health rather than the social construct of race alone, and that understanding these relationships may inform interventions to reduce these disparities in order to promote optimal maternal and infant health.

Acevedo-Garcia et al. have put forth the Child Opportunity Index (COI) to be better able to examine the plethora of community resources potentially available to foster healthy children’s development [31]. The COI capitalizes on a wide array of community-level measures available in open-source datasets to examine the complexities of resources as they relate to child and adult health. Analyses show that the overall COI is strongly correlated with measures of intergenerational economic mobility from the Opportunity Atlas and measures of health and life expectancy [31]. Additionally, a recent study found that the COI related to young children’s school readiness across a state, and the access to and benefit from these resources varied by child race/ethnicity [18]. Using the COI combined with maternal characteristics and race/ethnicity in our study, then, will allow for examination of all these factors simultaneously as they relate to the likelihood of breastfeeding.

### Purpose

The purpose of this study is to examine the association between community resources and any as well as exclusive breastfeeding among mothers of newly born infants, and whether this varies based on maternal race/ethnicity and country of birth. We aim to answer the following questions:

1. After accounting for maternal socio-demographics, are community resources (as defined by the COI) associated with the likelihood of a mother initiating and exclusively breastfeeding?
2. How does this association vary by maternal race/ethnicity and country of birth (U.S. or non-U.S. born) status?

## Method

### Sample

We used nationally representative data from the Study of Attitudes and Factors Effecting Infant Care (SAFE), which surveyed U.S. mothers immediately after an inf ant birth and when the infant was two to six months of age [34]. The SAFE Study used a stratified, two-stage, clustered design to obtain a nationally representative sample of mothers of infants, while oversampling Hispanic and non-Hispanic (NH) Black mothers. The SAFE study sample included mothers who spoke English or Spanish and were enrolled across 32 US birth hospitals between January 2011 and March 2014. Sample descriptives are in Table 1.

**Table 1:** Full sample characteristics

Characteristic	n (%)	Weighted Percent (95%CI)	US Vital Statistics (2011–2013) %
**Age at survey**			
8–11 weeks	2026 (61.4)	62.9 (59.4–66.4)	*
12–15 weeks	564 (17.1)	17.0 (15.5–18.5)	*
16–19 weeks	323 (9.8)	9.4 (7.6–11.2)	*
20+ weeks	384 (11.6)	10.7 (9.0-12.4)	*
**Gender**			
Male	1685 (51.2)	50.7 (49.0-52.4)	51.2
Female	1608 (48.8)	49.3 (47.6–51.0)	48.8
**Birth weight**			
<2500	202 (6.2)	5.7 (4.6–6.8)	8.0
2500+	3076 (93.8)	94.3 (93.2–95.4)	91.9
**Parity**			
1	1215 (37.0)	37.9 (35.3–40.5)	39.7
2	1095 (33.3)	33.7 (32.0-35.3)	31.5
3+	978 (29.7)	28.4 (25.7–31.2)	28.3
**Mother’s age**			
Less than 20	275 (8.3)	7.5 (4.8–10.3)	7.8
20 to 29	1788 (54.2)	52.2 (48.7–55.6)	51.6
30 or more	1234 (37.4)	40.3 (35.4–45.2)	40.6
**Race/ethnicity**			
NH-White	1275 (38.7)	52.3 (46.4–58.2)	54.1
NH-Black	828 (25.1)	12.9 (10.2–15.6)	14.8
Latiné/Hispanic	912 (27.7)	26.0 (20.9–31.1)	23.0
Other	281 (8.5)	8.8 (6.4–11.2)	8.0
**Mother’s Education**			
Less than HS	478 (14.6)	12.8 (8.7–16.9)	17.1
HS or GED	833 (25.4)	23.5 (20.6–26.5)	25.1
Some college	1041 (31.7)	30.8 (28.1–33.5)	29.0
College or more	932 (28.4)	32.9 (26.6–39.2)	28.8
**Region**			
Northeast	634 (19.2)	21.2 (2.5–39.9)	*
Midwest	496 (15.0)	12.8 (1.3–24.3)	*
South/Southeast	1383 (41.9)	41.5 (20.2–62.7)	*
West	784 (23.8)	24.5 (9.2–39.8)	*
**Household Income**			
Less than $20,000	1158 (35.1)	29.5 (24.7–34.3)	*
$20,000–49,999	838 (25.4)	24.8 (19.9–29.7)	*
$50,000 or more	577 (17.5)	19.8 (16.4–23.1)	*
Unknown	724 (22.0)	26.0 (20.0–32.0)	*
**Marital Status**			
Never Married	1454 (44.3)	37.7 (33.5–41.9)	*
Married	1658 (50.5)	57.0 (52.6–61.4)	*
Other	170 (5.2)	5.3 (4.0-6.6)	*

*Not available/applicable

### Measures

#### Maternal demographics

Mothers provided information about their age, race/ethnicity, birth country, education and employment status.

#### Breastfeeding behavior

Mothers were asked: “Over the LAST two weeks, what has your baby been drinking?”, with response options: “Only breastmilk”, “Mostly breastmilk”, “Equally breastmilk and formula”, “Mostly Formula”, “Only Formula” or “Other.” We created two outcome measures, “Any breastfeeding” (those who responded “Only breastmilk”, “Mostly breastmilk”, or “Equally breastmilk and formula”, or “Mostly Formula”) and “Exclusive breastfeeding” (those who responded “Only breastmilk”). Mothers in the category of “any breastfeeding” were compared to those who responded “Only Formula” or “Other”; those in the category of “exclusive breastfeeding” were compared to all other options.

#### Neighborhood resources

The Child Opportunity Index (COI) 2.0 is a census tract measure of neighborhood resources and conditions that matter for children’s healthy development. The COI 2.0 includes 29 indicators that measure neighborhood-based exposure to resources such as access and quality of early childhood education, green space, healthy food, toxin-free environments, and socioeconomic resources [35]. Tracts are nationally normed and then categorized into levels from 1 (*very low*) to 5 (*very high*) resourced neighborhoods.

### Statistical analyses

The first step in this study involved linking SAFE data with COI data. 3,297 participants completed the SAFE survey and were initially eligible for this analysis. In order to link to the COI, we used subject address data to obtain census level geographic data using the Census Geocoder tool (https://geocoding.geo.census.gov/geocoder/geographies/addressbatch?form). Of the 3,297 participants, we were able to match 2,942 with geographic data (89% of the sample). We then calculated the distribution of participants by level of COI for maternal age, race/ethnicity, education and employment, and child age at time of survey (Table 2).

**Table 2:** Sample characteristics, by Child Opportunity Index (COI) category (*n* = 2942)

		Overall COI Nationally Normed					
Characteristic	Overall	Very Low n (%)	Low n (%)	Moderate n (%)	High n (%)	Very High n (%)	p-value
Overall	2942	1047 (26.6)	692 (24.2)	474 (19.1)	461 (18.8)	268 (11.2)	
Mother’s age > 25	2000 (69.7)	589 (54.1)	444 (63.3)	330 (70.2)	390 (85.0)	247 (94.2)	< 0.0001
**Maternal race/ethnicity/nativity**							< 0.0001
US-born white	1095 (50.4)	152 (25.8)	251 (49.2)	253 (63.7)	270 (63.3)	169 (67.6)	
US-born Black	617 (10.5)	391 (23.9)	118 (8.3)	53 (4.8)	37 (4.3)	18 (3.6)	
Non-US-born Black	127 (2.4)	64 (4.1)	23 (1.7)	18 (2.2)	13 (1.5)	9 (1.5)	
US-born Hispanic	383 (12.8)	173 (19.4)	113 (15.8)	44 (8.6)	38 (8.6)	15 (4.7)	
Non-US-born Hispanic	424 (13.0)	202 (20.2)	128 (17.8)	49 (8.6)	32 (6.0)	13 (4.5)	
Other	296 (11.0)	65 (6.7)	59 (7.3)	57 (12.1)	71 (16.3)	44 (18.1)	
**Mother’s Education**							< 0.0001
Less than HS	416 (12.5)	263 (26.3)	96 (14.2)	35 (6.1)	21 (4.4)	1 (0.3)	
HS or GED	733 (22.7)	362 (34.0)	201 (28.5)	105 (22.6)	48 (10.0)	17 (4.7)	
Some college	938 (31.1)	319 (29.4)	261 (36.7)	161 (33.9)	151 (31.6)	46 (16.9)	
College Graduate	563 (21.8)	81 (7.8)	95 (14.8)	124 (25.6)	146 (32.6)	117 (45.7)	
Graduate School	292 (12.0)	22 (2.5)	39 (5.9)	49 (11.8)	95 (21.4)	87 (32.3)	
**Employed**	1694 (59.3)	490 (47.5)	381 (54.5)	306 (62.9)	325 (70.4)	192 (72.8)	< 0.0001
**Child age**							< 0.0001
8–11 weeks	1823 (63.6)	595 (56.4)	417 (61.3)	288 (64.1)	315 (68.4)	208 (76.6)	
12–15 weeks	492 (16.7)	180 (18.8)	125 (17.0)	82 (15.4)	68 (15.8)	37 (14.8)	
16–19 weeks	292 (9.4)	119 (11.1)	75 (11.4)	46 (9.2)	38 (7.5)	14 (5.0)	
20+ weeks	335 (10.3)	153 (13.7)	75 (10.3)	58 (11.3)	40 (8.4)	9 (3.7)	

Numbers in parentheses are weighted percentages adjusting for sampling design

To answer our research questions, we used multivariable logistic regression to examine the association between COI with any and exclusive breastfeeding. We present two models, first adjusting only for child age at survey and child sex, and a second model that adds the following maternal demographic characteristics: maternal age, self-identified race/ethnicity, employment, and education. To formally test for modification of the COI association by race/ethnicity, we tested for significant interaction between the COI and race/ethnicity in the fully adjusted model. We also performed separate analyses of the COI association by race/ethnicity. All analyses accounted for the cluster sample design by using appropriate SAS procedures for complex survey designs. Analyses were weighted to account for sampling probabilities and to reflect the national joint distributions of maternal age and race/ethnicity. Analyses were performed in SAS 9.4 (SAS Institute Inc.).

## Results

Data from 2,942 mothers indicated 60% engaged in any breastfeeding and 31% in exclusive breastfeeding at the two- to six-month postpartum follow-up (Table 3). The sample weighted distribution of the community resource levels as defined by the COI was as follows: very low (27%), low (24%), moderate (19%), high (19%), and very high (11%). Those living in higher COI neighborhoods were substantially more likely to be older than 25, white, have a college education or higher, and be employed. For example, 48% of mothers in the very low resourced communities, compared to 73% in the very high resourced communities, were employed.

**Table 3:** Associations between COI, any breastfeeding, and exclusive breastfeeding odds ratios and 95% confidence intervals from multiple logistic regression analysis, *n* = 2942

Characteristic	Overall n (%)	Weighted Percent Breastfeeding n (%)	OR for breastfeeding Adjusted for child age, sex	OR for breastfeeding Adjusted for child age, sex and Maternal Demographics
For any breastfeeding	2942	1721 (60.4)		
**Overall COI, nationally normed**				
Very Low	1047 (26.6)	530 (51.2)	REF	REF
Low	692 (24.2)	386 (56.1)	1.2 (0.9,1.7)	1.1 (0.8,1.4)
Moderate	474 (19.1)	273 (57.8)	1.3 (0.9,1.8)	1.0 (0.8,1.4)
High	461 (18.8)	325 (70.8)	2.2 (1.5,3.3)	1.4 (1.0,2.1)
Very High	268 (11.2)	207 (78.8)	3.3 (2.3,4.7)	1.6 (1.1,2.3)
For exclusive breastfeeding	2942	821 (30.6)		
**Overall COI, nationally normed**				
Very Low	1047 (26.6)	187 (20.2)	REF	REF
Low	692 (24.2)	172 (25.7)	1.3 (1.0,1.8)	1.0 (0.8,1.4)
Moderate	474 (19.1)	136 (28.4)	1.5 (1.1,2.2)	1.0 (0.7,1.4)
High	461 (18.8)	190 (42.0)	2.7 (1.8,4.1)	1.5 (1.0,2.3)
Very High	268 (11.2)	136 (50.7)	3.6 (2.6,4.8)	1.7 (1.3,2.3)

Weighted analysis accounting for sampling design

Demographics– maternal age, race, employment, and education

The likelihood of breastfeeding increased as the neighborhood COI increased. Compared to mothers residing in the lowest resourced neighborhoods, after accounting for maternal individual characteristics, those residing in the highest resourced neighborhoods had a significantly greater likelihood of breastfeeding (aOR 1.63; 95% CI 1.14, 2.33 and 1.69; 95% CI 1.26, 2.26; for any and exclusive breastfeeding at follow-up, respectively). Further, differences were examined by maternal race/ethnicity and are presented in Table 4. While access to higher resourced neighborhoods differed by race/ethnicity, it did not significantly moderate the association between COI and breastfeeding. When looking within race/ethnicity groups by whether mothers were born in or outside the US in the interaction models, however, a different pattern emerged. Although cell sizes become very small in this analysis, findings indicate that COI is not related to non-US born Black and Hispanic mothers’ rates of breastfeeding, while it is with US born Black and Hispanic (and white) mothers. For US born Hispanic mothers, the association with COI is most apparent for exclusive breastfeeding.

**Table 4:** Breastfeeding and COI stratify by race/ethnicity and birth country

	n (%)	Weighted Percent Breastfeeding n (%)	OR for breastfeeding Adjusted for child age, sex	OR for breastfeeding Adjusted for child age, sex and Maternal Demographics
**US born White**	1095	640 (58.2)		
For any Breastfeeding				
Overall COI, nationally normed				
Very Low	152 (13.6)	61 (41.3)	REF	REF
Low	251 (23.6)	128 (50.1)	1.4 (0.9,2.2)	1.1 (0.8,1.6)
Moderate	253 (24.2)	139 (54.5)	1.7 (1.1,2.4)	1.1 (0.8,1.5)
High	270 (23.6)	181 (66.3)	2.7 (1.6,4.5)	1.2(0.8,1.9)
Very High	169 (15.0)	131 (79.5)	5.1 (3.3,8.0)	1.8 (1.1,2.7)
For exclusive breastfeeding	1095	406 (35.8)		
Overall COI, nationally normed				
Very Low	152 (13.6)	36 (25.1)	REF	REF
Low	251 (23.6)	71 (27.1)	1.1 (0.7,1.9)	0.9 (0.5,1.5)
Moderate	253 (24.2)	80 (28.3)	1.2 (0.7,2.0)	0.8 (0.5,1.4)
High	270 (23.6)	126 (45.5)	2.4 (1.2,4.7)	1.3(0.8,2.4)
Very High	169 (15.0)	93 (55.8)	3.4 (2.1,5.7)	1.6 (1.0,2.6)
**US born Black**				
For any breastfeeding	617	241 (40.4)		
Overall COI, nationally normed				
Very Low	391 (60.6)	147 (37.9)	REF	REF
Low	118 (19.1)	46 (39.9)	1.1 (0.7,1.8)	1.0 (0.6,1.6)
Moderate	53 (8.8)	21 (39.6)	1.1 (0.6,2.2)	0.9 (0.5,1.7)
High	37 (7.7)	15 (46.8)	1.5 (0.7,3.1)	1.0 (0.5,2.2)
Very High	18 (3.8)	12 (71.6)	3.9 (1.3,11.1)	2.3 (0.7,7.9)
For exclusive breastfeeding	617	85 (14.3)		
Overall COI, nationally normed				
Very Low	391 (60.6)	46 (12.4)	REF	REF
Low	118 (19.1)	17 (13.7)	1.2 (0.5,2.7)	1.1 (0.4,2.5)
Moderate	53 (8.8)	7 (10.3)	0.9 (0.4,2.0)	0.8(0.4,1.8)
High	37 (7.7)	9 (30.1)	3.8 (1.5,9.5)	2.9 (1.2,7.0)
Very High	18 (3.8)	6 (24.6)	2.0 (0.8,4.9)	1.1 (0.5,2.6)
**Non-US born Black**				
For any breastfeeding	127	106 (83.0)		
Overall COI, nationally normed				
Very Low	64 (45.8)	56 (87.1)	REF	REF
Low	23 (17.3)	15 (64.2)	0.2 (0.1,0.5)	0.1 (0.0,0.5)
Moderate	18 (17.6)	14 (75.1)	0.3 (0.1,0.9)	0.1 (0.0,0.3)
High	13 (12.2)	13 (100)	--	--
Very High	9 (7.2)	8 (93.5)	1.9 (0.2,21.0)	0.4 (0.0,3.0)
For exclusive breastfeeding	127	26 (20.9)		
Overall COI, nationally normed				
Very Low	64 (45.8)	12 (22.9)	REF	REF
Low	23 (17.3)	6 (26.8)	1.3 (0.5,3.6)	1.3 (0.4,3.8)
Moderate	18 (17.6)	2 (10.4)	0.4 (0.1,1.9)	0.3 (0.0,2.9)
High	13 (12.2)	1 (4.1)	0.1 (0.0,1.7)	0.1 (0.0,2.7)
Very High	9 (7.2)	5 (48.3)	2.7 (0.5,14.9)	2.2 (0.3,15.4)
**US born Hispanic**	383	216 (58.7)		
For any breastfeeding				
Overall COI, nationally normed				
Very Low	173 (40.4)	83 (50.3)	REF	REF
Low	113 (29.9)	66 (58.4)	1.4 (0.8,2.5)	1.3 (0.7,2.4)
Moderate	44 (12.9)	29 (68.3)	2.2 (0.8,5.7)	1.7 (0.6,5.3)
High	38 (12.6)	28 (73.9)	2.5 (1.1,5.9)	2.0 (0.8,5.1)
Very High	15 (4.1)	10 (66.8)	2.2 (0.8,6.4)	1.5 (0.4,5.6)
For exclusive breastfeeding	383	100 (29.0)		
Overall COI, nationally normed				
Very Low	173 (40.4)	34 (22.4)	REF	REF
Low	113 (29.9)	26 (23.5)	1.1 (0.7,1.8)	1.0 (0.6,1.7)
Moderate	44 (12.9)	15 (35.9)	2.0 (0.9,4.5)	1.7 (0.7,4.1)
High	38 (12.6)	16 (46.1)	3.0 (0.9,9.5)	2.4 (0.7,8.4)
Very High	15 (4.1)	9 (59.8)	5.5 (2.1,14.8)	4.7 (1.5,14.5)
**Non-US born Hispanic**				
For any breastfeeding	424	313 (72.0)		
Overall COI, nationally normed				
Very Low	202 (41.4)	145 (70.9)	REF	REF
Low	128 (33.3)	91 (69.7)	0.8 (0.4,1.6)	0.8 (0.4,1.7)
Moderate	49 (12.7)	37 (66.3)	0.7(0.3,2.1)	0.8(0.3,2.5)
High	32 (8.8)	28 (84.6)	1.9 (0.5,7.1)	1.9 (0.5,7.2)
Very High	13 (3.9)	12 (93.0)	4.5 (0.7,27.7)	5.4 (1.0,29.9)
For exclusive breastfeeding	424	108 (24.7)		
Overall COI, nationally normed				
Very Low	202 (41.4)	44 (19.4)	REF	REF
Low	128 (33.3)	35 (27.5)	1.4 (0.7,2.7)	1.3 (0.7,2.6)
Moderate	49 (12.7)	13 (25.1)	1.3 (0.4,4.1)	1.2 (0.3,4.1)
High	32 (8.8)	13 (41.8)	2.5 (1.0,6.3)	2.5 (1.0,6.4)
Very High	13 (3.9)	3 (16.0)	0.6 (0.1,3.5)	0.7 (0.1,4.8)
**Other**				
For any breastfeeding	296	205 (73.1)		
Overall COI, nationally normed				
Very Low	65 (16.3)	38 (57.6)	REF	REF
Low	59 (16.0)	40 (75.1)	2.1 (0.9,5.0)	1.3(0.5,3.8)
Moderate	57 (21.2)	33 (65.3)	1.4 (0.6,3.6)	0.6 (0.2,1.8)
High	71 (28.0)	60 (85.0)	3.9 (1.6,9.7)	1.6 (0.6,4.3)
Very High	44 (18.5)	34 (76.1)	2.2 (1.1,4.4)	0.6 (0.2,1.5)
For exclusive breastfeeding	296	96 (33.7)		
Overall COI, nationally normed				
Very Low	65 (16.3)	15 (24.1)	REF	REF
Low	59 (16.0)	17 (29.1)	1.2 (0.4,3.7)	0.7 (0.2,2.7)
Moderate	57 (21.2)	19 (36.4)	1.9 (0.7,5.1)	1.1 (0.4,3.3)
High	71 (28.0)	25 (33.3)	1.4 (0.6,3.4)	0.6(0.3,1.7)
Very High	44 (18.5)	20 (43.5)	2.2 (0.8,5.8)	0.8 (0.3,2.4)

Weighted analysis accounting for sampling design

Demographics– maternal age, race, employment, and education

## Discussion

In this study we aimed to examine the association of neighborhood resources and breastfeeding. Findings showed that mothers living in communities where more resources exist to facilitate children’s development, as defined by the COI, had a higher likelihood of breastfeeding, and that access to these higher resourced communities varied systematically by race/ethnicity. That is, mothers of color were more likely to live in low resourced communities. Although the association between COI and breastfeeding was not moderated by race/ethnicity, patterns changed when within group differences were examined. Taken together, these findings shed light on the importance of examining patterns of breastfeeding initiation and duration in more nuanced ways that capture context and parental characteristics. Key findings and their significant contributions are described in more detail below.

### Neighborhood resources matter for infant feeding practice

Compared to mothers residing in the lowest resourced neighborhoods, those residing in the highest resourced neighborhoods as indicated by the COI score had a significantly greater likelihood to initiate breastfeeding. This association existed after accounting for individual maternal characteristics previously shown to relate to breastfeeding. Given that breastfeeding has significant and long-lasting effects on cognition, behavior, and mental health in children [7], this finding provides new insights on another mechanism to consider in fostering healthy child development.

It is possible that neighborhoods matter because of social connections given that informal education, support networks, and context shape norms and attitudes as well as an understanding of the importance of breastfeeding. For example, in a recent study of 3,297 mother-infant dyads, positive attitudes and social norms mediated the association between maternal education and breastfeeding [36]. Mothers who have personal social networks in which breastfeeding is the norm are more likely to do so [37]. Thus, prior research coupled with our findings emphasize the importance of neighborhood opportunities–including resources such as access and quality of early childhood education and healthy environments, but also social support networks, to promote healthy childhood development. Future research should dig more deeply into these relationships between various neighborhood resources to better understand their impacts on breastfeeding initiation and child development.

### The role of race/ethnicity and country of birth in access to resources

Access to more resourced neighborhoods varied considerably by race/ethnicity; mothers who identified as Black or Hispanic were over-represented in lower-resourced communities, whereas those living in higher-resourced neighborhoods were substantially more likely to be white. This finding aligns with prior work using the COI index that found an over-representation of marginalized children in lower resourced neighborhoods [18]. It also builds on prior work that has identified large racial/ethnic disparities in breastfeeding initiation and duration [2] to emphasize how the context in which parents and families live likely contribute to these disparities. Further, mothers who are white, employed, and/or more highly educated are more likely to have larger personal social networks– and these networks are more likely to embrace ideas such as breastfeeding [38]. Thus, inequitable access to neighborhood resources and the associated social supports should be addressed through policy and practice to facilitate child development across racial and ethnic groups.

While findings did not demonstrate that race/ethnicity significantly moderated the association between neighborhood opportunities and breastfeeding initiation across all groups, within group differences were present. Specifically, findings indicate that neighborhood resources were not related to breastfeeding rates among Black and Latiné/Hispanic mothers born outside of the US, but were related to rates for US born Black, Latiné/Hispanic and white mothers. Similar to the study by Safon and colleagues, this finding suggests that a parents’ country of birth may serve as a protective factor for breastfeeding initiation patterns in the US [15]. However, this finding also suggests that parents socialized in the US as compared to other countries may not have access to the resources and knowledge to support breastfeeding, particularly if they reside in a neighborhood with fewer resources.

### Limitations

There are several limitations that affect our findings. First, we did not collect transgender or non-binary gender demographic data. These parents may face challenges in accessing neighborhood resources that cis-gender women do not face. In our future work on this topic, we plan to include more gender demographic data to examine how these relationships differ across gender identities.

Additionally, although the study sample size was large, cells became very small when disaggregating the data in regard to country of birth. This limited the exploration of neighborhood resources and breastfeeding rates across specific countries. Building from the study results indicating differences for participants born in the US and those born outside of the U.S., future studies should examine differences across various countries with larger samples.

Finally, the data used in this study were not collected with the explicit purpose of addressing this research question. Thus, while the results provide important information about neighborhood resources and breastfeeding, other support characteristics were not included. Future work should continue to widen the types of supports and barriers experienced by parents to better understand the multi-systemic influences on breastfeeding.

## Conclusion

This study highlighted important relationships between neighborhood resources and breastfeeding practices. Furthermore, our study found Black and Latiné/Hispanic parents were more likely to reside in lower resourced neighborhoods, highlighting persistent inequities present in the US. This shows that even health behaviors we often associate with individual choice are also connected to the environment within which they are made. Thus, understanding how neighborhood resources relate to individual health behaviors such as breastfeeding requires a complex examination of the multiple systems and contextual factors that shape health.

## Data Availability

De-identified individual participant data (including data dictionaries), study protocols, the statistical analysis plan, and the informed consent form will be made available upon publication to researchers who provide a methodologically sound proposal for use in achieving the goals of the approved proposal. Proposals should be submitted to Jennifer LoCasle-Crouch, locasalecrj@vcu.edu.
